# The patterns of inbreeding depression in food-deceptive *Dactylorhiza* orchids

**DOI:** 10.3389/fpls.2024.1244393

**Published:** 2024-03-25

**Authors:** Ada Wróblewska, Beata Ostrowiecka, Emilia Brzosko, Edyta Jermakowicz, Izabela Tałałaj, Paweł Mirski

**Affiliations:** Faculty of Biology, University of Bialystok, Białystok, Poland

**Keywords:** *Dactylorhiza fuchsii*, *Dactylorhiza incarnata* var. *incarnata*, *Dactylorhiza majalis*, fruit set, inbreeding depression, in vitro asymbiotic seed germination

## Abstract

**Introduction:**

Inbreeding depression (ID) in food-deceptive plants have been reported previously, however, it has not been often proven that selfed seeds germinate better than outbred ones or that selfing affects ID. To resolved these issues, food-deceptive related *Dactylorhiza majalis*, *D. incarnata* var. *incarnata* and *D. fuchsii* orchids were investigated.

**Methods:**

Hand pollination treatments and control pollination were conducted. Fruit set, number of seeds per fruit, seed length, number of well-developed seeds per fruit, and proportion of *in vitro* asymbiotic germination seeds, were analyzed in relation to inflorescence levels and used as fitness indicators for these orchids. The ID and pollen limitation were measured.

**Results:**

The lowest ID (*δ* = −1.000) was in *D. majalis*, and present in combination with a high pollen limitation in its populations. *D. fuchsii* showed higher ID (*δ* = 0.366), and *D. incarnata* var. *incarnata* weak ID (*δ* = 0.065), although ID varied between its fitness components. The seed number per fruit differed significantly between the treatments and the inflorescence levels in treatments.

**Discussion:**

This study emphasizes that the breeding system rather than the flower position on the inflorescence shaped the quality and quantity of reproductive output. The ID and its effect on germination of food-deceptive orchid seeds undoubtedly played an important role.

## 1 Introduction

Inbreeding depression (ID) is a mechanism that is defined as the reduction in the fitness of offspring resulting from an interbreeding between related individuals (Barrett & Harder, 2017). The effects of ID have usually been studied in relation to the mating systems, population size, area of geographical distribution, and their consequences on different developmental plant life stages (i.e., seeds, juveniles, and adult stages) (Glaettli & Goudet, 2006; Angeloni et al., 2011; Valtueña et al., 2014; Blambert et al., 2016). Angeloni et al. (2011), using meta-analysis of 116 studies and 107 plants, found that ID was significant for all life history stages and reproductive traits. In their study, ID increased with population size and varied depending on the method of measuring plant fitness. They also concluded, that self-compatible plant species are much more prone to inbreeding than self-incompatible species, and often to display ID. This can contribute to a faster removal of deleterious alleles in different life stages. They also explained that significant ID was found for all the life stages, but the lowest ID in the early life stage (i. g. germination) than in the late life stages for self-compatible species can occur.

From another point of view, the study by Baskin & Baskin (2015) on 743 germination cases has showed that outbred seeds can germinate better, equal (in 50.1% of cases), or less well than inbred seeds. The authors recommended improving the germination procedure in the natural environment, but there is no doubt that this procedures requires further investigation. Mechanisms associated with ID, such as a lower germination rate and low juvenile survival, as well as a decline in the growth fitness of progeny derived from selfing, promote undoubtedly the evolution of outcrossing (Smithson, 2006).

The mating system has frequently been emphasized as the best predictor of ID (Johnson & Nilsson, 1999; Juillet et al., 2006). In Orchidaceae, in which approximately one-third of all known orchids implement deception, outcrossing is exhibited as the main mating system (Ackerman, 1986; Dafni, 1987; Nilsson, 1992; Jersáková et al., 2006). Husband & Schemske (1996) reported that genetic load is observed in outcrossing plants to favor the evolution if inbreeding avoidance. They showed empirical evidences that outcrossing species have significantly greater ID at seed viability than selfing species. Therefore, ID may be considered as a mechanism favoring the evolution and maintenance of food deception strategy in orchids. Then, Smithson (2006) compiled ID from the literature based on the various life stages within nectarless orchids (*δ* = -0.064 – 0.713) and these wild spectrum of values did not fully support the predictions and experimental data of Husband and Schemske (1996). However, the earlier scarce surveys of food-deceptive, and self-compatible orchids evaluated that the fruit set was the effect of different mating system i.e. outcrossing, selfing, or mix-mating. In this way, the mating systems can contribute to the different effects of reproductive output and ID (Johnson & Nilsson, 1999; Smithson, 2006; Tremblay et al., 2005; Kropf & Renner, 2008; Ostrowiecka et al., 2019).

Our study focused on *Dactylorhiza* taxa, which are food-deceptive orchids with no rewards for their pollinators (Claessens and Kleynen, 2011). More published studies of ID have analyzed an early life stage in this group of plants, but there are still often contradictory results. In *Dactylorhiza* genus, only two taxa, *D. sambucina* and *D. praetermissa*, have been examined for ID (Ferdy et al., 2001; Juillet et al., 2006). Depending on different developmental life stages of these plants (e. g. seeds, germination, or juvenile and adult) and type of crosses (outcrossing or selfing) both strong and weak ID were recognized. The negative correlation between fitness indicators and the position of the fruit on *D. praetermissa* inflorescence suggested that resource might be limited, and selective abortion might be important. Further, ID may vary through ontogeny, and the total amount of ID suffered by an inbred individual is a product of reduced fitness at all life stages. In this context, in ID studies of food-deceptive *Dactylorhiza* taxa it is important to consider their current mating system, and to compare fitness component traits at different life stages in natural pollinated, inbred, and outbred individuals (Carr & Duasch, 1996; Husband & Schemske, 1996). Therefore, we focused on three *Dactylorhiza* taxa i.e. *D. majalis*, *D. incarnata* var. *incarnata*, and *D. fuchsii*. They are terrestrial, long-lived, self-compatible, and tuberous perennial orchids that reproduce either by seeds or (rarely) vegetatively (Vakhrameeva et al., 2008; Claessens and Kleynen, 2011; Ostrowiecka et al., 2019). They produce a single multi-flowered inflorescence and flowers with short spurs (Naczk et al., 2018). The flower number on inflorescence differs between taxa, and they open from bottom to top sequentially but very quickly or all flowers on an inflorescence can be open simultaneously (Ostrowiecka et al., 2019). Pollination occurs by different taxonomical groups of insects (Hymenoptera, Diptera, and/or Coleoptera), which can promote cross-pollination and/or geitonogamy (Hedrén & Nordström, 2009; Ostrowiecka et al., 2019; Wróblewska et al., 2019). *Dactylorhiza majalis* blooms first from the beginning of May to the beginning of June. The flowering period of *D. incarnata* and *D. fuchsii* occurs between June and July (Ostrowiecka et al., 2019), and the fruits occur from the end of July. The variable fruit set ranges from 7.4% to 77.5% in three *Dactylorhiza* taxa (Vallius et al., 2004; Kindlmann & Jersáková, 2006; Claessens and Kleynen, 2011). Pollen limitation has also been observed in these plants and has been recognized as an important phenomenon that shapes the quality and quantity of the fruit set and seeds (Kropf and Renner, 2008). Molecular markers such as cpDNA (trnL, trnF and psbC–trnK), internal transcribed spacer (ITS) sequences, and flow cytometry data confirmed the taxonomic status of the studied three orchids (Wróblewska et al., 2019).

Here, the second key issue in investigation ID patterns of three *Dactylorhiza* taxa is phylogenetic relationship between them. *Dactylorhiza majalis* is allotetrapolyploid, and has survived in central European glacial refugia during the last ice age (Balao et al., 2016), or formed probably at different times in the recent half of the Quaternary (Brandrud et al., 2020; Hawranek, 2021). Clo (2022) investigated the effect of polyploidy on the evolution of ID using meta-analysis, and hypothesized that ID should be lower in allopolyploids compared to diploids progenitors. This meta-analysis showed theoretical expectations that due to an initial bottleneck, the masking of deleterious mutations, and/or a slower increase in homozygosity during selfing events, (new)polyploid lineages benefit from a strong decrease in ID (se also e. g. Otto & Whitton, 2000; Clo, 2022). However, empirical studies on the relationship between allopolyploidy and ID have still produced conflicting results (Johnston and Schoen, 1996; Rosquist, 2001; Pannell et al., 2004; Barringer & Geber, 2008; Vandepitte et al., 2011). In resolving these above-mentioned issues and to bridge the knowledge gap between food-deception strategy and mating systems in closely related diploid and polyploid *Dactylorhiza* orchids, we analyzed the various fitness traits (i.e., fruit set, quantity and quality of seeds, and germination) over the life stage categories following control pollination, self and outcross hand pollinations to determine (1) how mating system shapes the magnitude of ID at these fitness components, (2) how the flower position along the inflorescence affects reproductive success and ID, and (3) what are the fitness traits in which ID is the more pronounced.

## 2 Material and methods

### 2.1 Study sites

**Figure 1:**
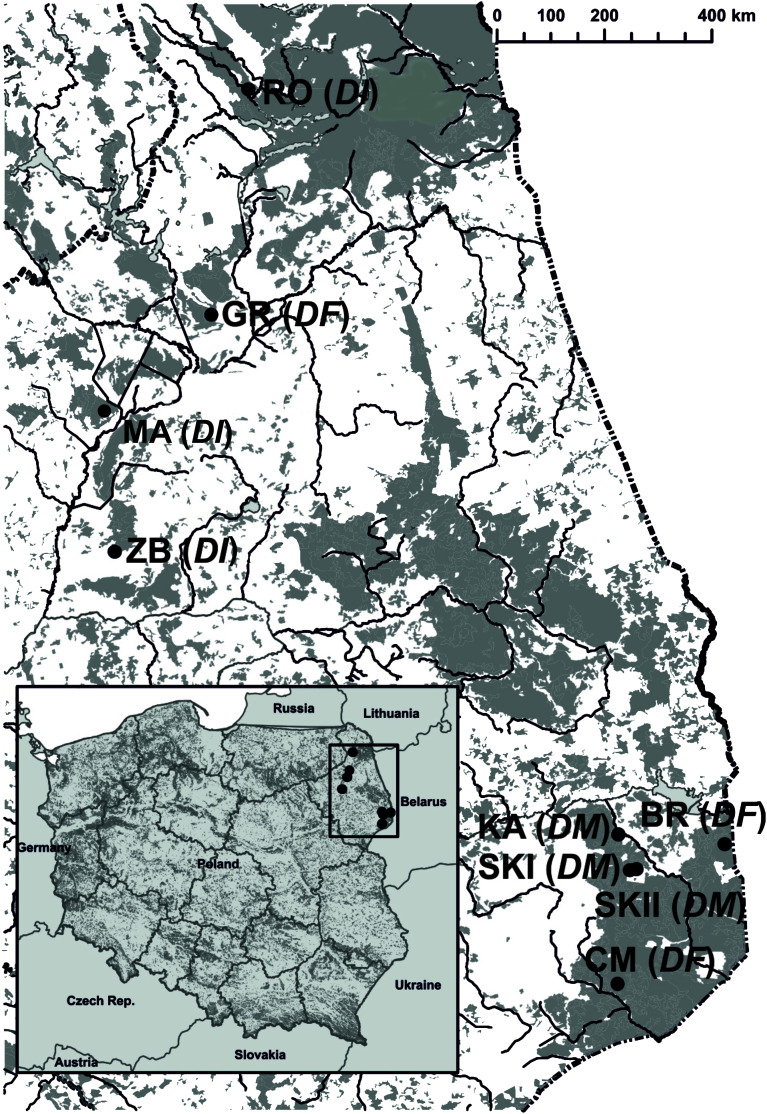
Localities of nine *Dactylorhiza* populations in north-eastern Poland. *D. majalis* (*DM*), KA, SKI, and SKII, *D. incarnata* var. *incarnata* (*DI*), ZB, MR, and RO, *D. fuchsii* (*DF*) CM, BR, and GR.

This two-year study was conducted from May to July across three populations each of *D. majalis* (KA, 2014-2015; SKI, 2015-2016; and SKII, 2016-2017), *D. incarnata* var. *incarnata* (ZB, 2014-2015; RO and MR, 2015-2016), and *D. fuchsii* (BR, CM, and GR, 2015-2016), located in northeastern Poland (**Supplementary Table S1**; **Figure 1**). *Dactylorhiza majalis* grows in wet meadows, cohabitating with abundant entomophilous and rewarding plants, comprising approximately 20% of the herb layer. The abundance of *D. majalis* varied, with around 120–200 (to ca. 1,000) flowering individuals (**Supplementary Table S1**). The three *D. incarnata* var. *incarnata* populations varied in sizes ranging from 35 to 200 flowering plants (**Supplementary Table S1**). These populations inhabited sedge communities in the Biebrza Valley and Rospuda Valley, with a rewarding plant species cover of about 10%. *Dactylorhiza fuchsii* was found in open hornbeam forests in the Białowieża Primeval Forest and nearby areas and one population in the Biebrza Valley (84–193 flowering plants), all with a low density of rewarding plants.

### 2.2 Open and hand pollination experiments

Over a two-year period (2014 to 2017), open and hand pollination experiments were conducted on three *Dactylorhiza* taxa during peak flowering, when most flowers were open. Open pollination treatments (n = 5 inflorescences per population/year, 90–340 flowers per population/year, **Supplementary Table S1**) and four hand pollination experiments were carried out on bagged inflorescences, during the optimum of flowering (almost all freshly flowers on the inflorescences were opened). For the hand pollination treatments, we employed three treatments on nylon mesh bag-enclosed inflorescences: (1) spontaneous self-pollination to assess autogamy (n = 2–5 inflorescences per population/year, 20–154 flowers per population/year, **Supplementary Table S1**), (2) induced self-pollination to determine self-compatibility (using pollen from the same flower) (n = 2–5 inflorescences per population/year, 37–203 flowers per population/year, **Supplementary Table S1**), and (3) geitonogamy (using pollen from another flower on the same plant) (n = 1–5 inflorescences per population/year, 37–203 flowers per population/year, **Supplementary Table S1**). Additionally, (4) induced xenogamy (cross-pollination with pollen from different plants within the same population, approximately 3 m away) was performed (n = 5 inflorescences per population/year, 76–177 flowers per population/year, **Supplementary Table S1**). All flowers for each treated plant were hand pollinated. After each treatment, the inflorescences were quickly rebagged. In total, 518 inflorescences and 9994 flowers were analyzed over two years across all treatments and taxa (**Supplementary Table S1**).

Pollination treatments were conducted in two stages: initially without division of the inflorescence, followed by targeted treatments on lower, middle, and upper sections of the inflorescence in each population to investigate the effect of flower position on fitness traits (fruit set, seed quantity and quality, germination). Flower allocation to each level was based on near-equal numbers. Statistical analyses of seed variability and the likelihood of *in vitro* asymbiotic germination to protocorm stage were performed for all hand pollination treatments, both divided and non-divided of inflorescence.

### 2.3 Fruit set, seed quantity and quality

Upon maturity, fruit set, total seed number per fruit, mean viable seed length (mm), seed categories based on embryo shape, and viable seeds germinating into protocorms on various asymbiotic media were measured. Seeds were immediately sown post-collection and evenly spread in Petri dishes marked with a grid (6 × 6 for *D. fuchsii* and *D. majalis*, 10 × 11 for *D. incarnata* var. *incarnata*). A random selection of 11 or 20 squares (confidence level of 0.02) was used for analysis, with a pseudorandom number generator implemented in the R environment (R 3.6/4.0 software, R Core Team, 2021). Categories included seeds with deformed embryos and well-developed globular embryos (Lemus et al., 2021). Sixty well-formed seeds per capsule were randomly chosen and measured using an OLYMPUS SZX2-ILLT stereomicroscope and MultiScan v.14.02 software.

For *in vitro* germination, seeds were stratified at 4°C for 12 weeks, surface sterilized in 5% NaOCl with Tween 80, and air-dried before sowing on Malmgren (1996) (*D. fuchsii*) and FAST (Lindén, 1980) (*D. majalis* and *D. incarnata* var. *incarnata*) media. Germination and protocorm development were assessed under a microscope after 40 days of incubation in the dark at 19–20°C. Total seed germination and protocorm development were assessed under a light microscope.

## 3 Data analysis

### 3.1 Fruit set and seed variability

Given the non-normal distribution of the data, traditional transformations (arcsin, log + 1, square root) were ineffective for ANOVA requirements. Therefore, Kruskal–Wallis tests and Dunn’s post-hoc tests with Bonferroni correction were employed to identify differences in fruit production, seed number per fruit, seed length, well-developed seed frequency per fruit, and *in vitro* germination rates between control and hand pollination treatments, and among flower positions on the inflorescence for each taxon. Variables included treatment types (control, selfing, cross-pollination), inflorescence levels (lower, middle, upper), and dependent factors (fruit set, seed number, seed length, well-developed seed proportion, *in vitro* germination rates). R 3.6/4.0 software was used for analysis, and standard errors were provided with mean values (R Core Team, 2021).

### 3.2 ID coefficient and pollen limitation

The ID coefficient *δ* was calculated from five reproductive traits (fruit set, seed number, seed length, well-developed seed proportion, *in vitro* germination), both for entire inflorescences and division of inflorescence on three levels, using formulas by Ågren and Schemske (1993) and Lande and Schemske (1985). The formula *δ* = 1 − (*W*s/*W*o) was used when *W*s < *W*o, and δ = (*W*o/*W*s) − 1 for *W*s > *W*o, where *W*s represents the average fitness of selfed progeny and *W*o represents that of outcrossed progeny. The estimation was carried out using data where *W*s denotes the average fitness of selfed progeny from autogamy and geitonogamy treatments and *W*o denotes the average fitness of manually outcrossed progeny (Lande and Schemske, 1985; Charlesworth & Charlesworth, 1987). Cumulative δ values were calculated incorporating the correlation among data sets, using the approach by Husband & Schemske (1996): *δ* = 1 − (*W*s_F_/*W*o_F_ x *W*s_NS_/*W*o_NS_ x *W*s_SL_/*W*o_SL_ x *W*s_DS_/*W*o_DS_ x *W*s_IV_/*W*o_IV_), where F is fruit set; NS is seed number per fruit; SL is seed length, DS is frequency of properly developed seeds per fruit, and IV is proportion *in vitro* asymbiotic germinated seeds to protocorm. The values of *δ* range from −1 to 1, where 0 signifies no ID, positive values indicate that the outcrossed offspring outperformed the inbred offspring, and negative values indicate the opposite (Charlesworth & Charlesworth, 1987).

Pollen limitation (PL) was calculated using the Larson and Barrett (2000) formula: PL = 1 – (control fruit set/cross-pollination fruit set), where PL values range from 0 (no pollen limitation) to 1 (maximum pollen limitation), and negative values indicate greater pollen receipt in naturally pollinated flowers (Lázaro and Traveset, 2006, González-Varo et al., 2009).

## 4 Results

### 4.1 Reproductive success and seed viability

**Figure 2:**
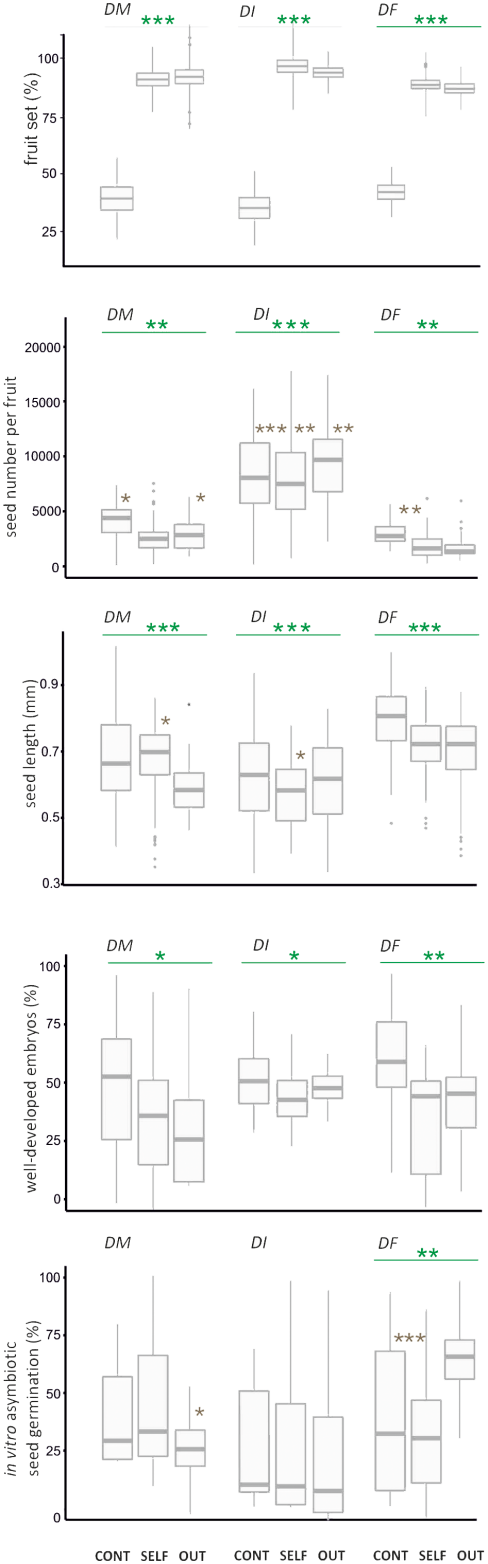
Fitness traits for fruit set quantity and quality, and germination of *Dactylorhiza majalis* (*DM*), *D. incarnata* var. *incarnata* (*DI*), and *D. fuchsii* (*DF*) seeds that were the effect of control pollination (natural pollination, CONT), selfing (SELF), and outcrossing (OUT). Dark green line, comparison between treatments; Brown asterisk, comparison between levels of inflorescence; *p < 0.05; **p < 0.001; ***p < 0.001. The box-and-whisker plot includes the mean (denoted by a horizontal bar in the box), standard error (denoted by a square), and standard deviation (denoted by the box).

There were significant differences in fruit set between control pollination and hand pollination treatments in the three Dactylorhiza taxa (**Figure 2**; **Supplementary Table S2**). In *D. majalis*, *D. incarnata* var. *incarnata*, and *D. fuchsii*, fruit set was 3 to 4 times lower in control pollination (35.7–44.0%) compared to hand pollination (85.0–90.2% for cross-pollination and 88.9–95.6% for self-pollination, **Figure 2**; **Supplementary Table S2**). No significant decrease in fruit set was observed from the lower to the upper position on the inflorescence for each *Dactylorhiza* species in both control and hand pollination treatments (**Figure 2**; **Supplementary Table S1**). Spontaneous autogamy was recorded in less than 1% of all studied flowers across the three *Dactylorhiza* taxa; hence, it was excluded from further analysis.

The seed number per fruit varied significantly between control and hand pollination treatments, and often between inflorescence levels within each taxon (**Figure 2**). In *D. majalis* and *D. fuchsii*, seed numbers per fruit were higher in control pollination than in both selfing and crossing experiments. In contrast, *D. incarnata* var. *incarnata* exhibited a higher number of seeds in outcrossing compared to selfing and control pollination (**Figure 2**, **Supplementary Table S2**). The lower part of the inflorescence produced a higher seed count per fruit than the upper part in control pollination across the three taxa (**Supplementary Table S2**).

Significant variations in seed length and the number of seeds with well-developed embryos were observed only between the different orchid treatments (**Table S2**). Seeds from control pollination showed a higher frequency of well-developed embryos than those from selfing and outcrossing in all *Dactylorhiza* taxa (**Figure 2**).

No significant differences were observed in *in vitro* asymbiotic seed germination between control and hand pollination treatments in *D. majalis* and *D. incarnata* var. *incarnata* (**Figure 2**; **Supplementary Table S2**). However, a significant difference was noted in the proportion of *in vitro* germinated seeds among all treatments in *D. fuchsii* (**Figure 2**). Notably, *in vitro* asymbiotic seed germination varied significantly between inflorescence levels in control pollination for *D. fuchsii* and in cross-pollination for *D. majalis* (**Supplementary Table S2**). The frequency of *in vitro* seed germination from the control fruit set was comparable to that of *in vitro* germinated seeds from selfing in *D. fuchsii* (**Figure 2**).

### 4.2 ID and pollen limitation

**Figure 3:**
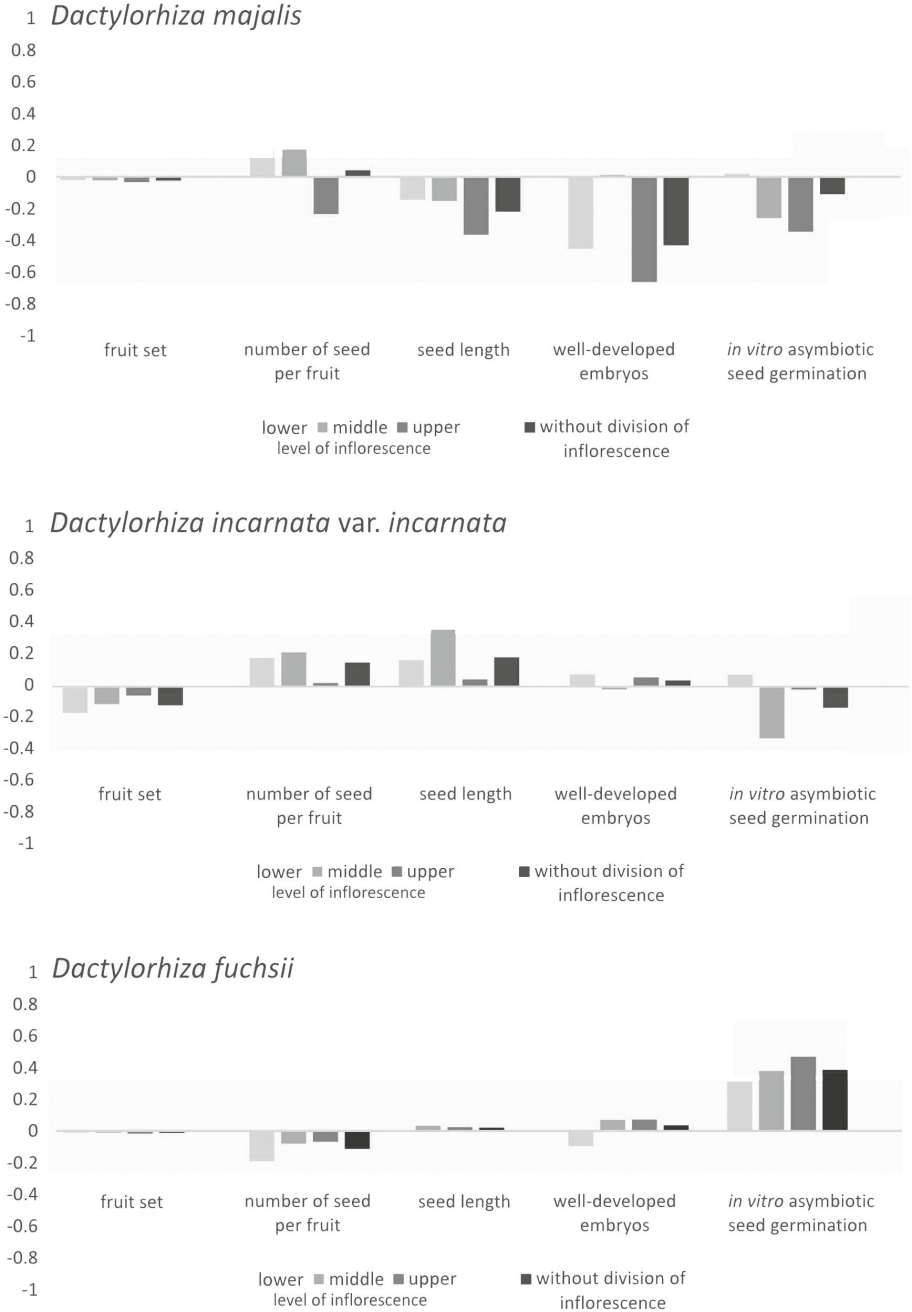
Distribution of inbreeding depression (*δ*) in fruit set, seed number per fruit, seed length, frequency of well-developed embryos in seeds, and proportion of *in vitro* asymbiotic seed germination of *Dactylorhiza majalis*, *D. incarnata* var. *incarnata*, and *D. fuchsii*, with division on three levels and without division of inflorescence (see text, Material and Methods).

The three *Dactylorhiza* taxa exhibited varying levels of ID. The cumulative ID at the taxa level was negative and lowest in *D. majalis* (δ = −1.000), nearly close to zero (δ = 0.065) in *D. incarnata* var. *incarnata*, and moderately positive in *D. fuchsii* (δ = 0.366). Differences in ID were observed between species for all fitness parameters and across inflorescence levels (**Figure 3**). *Dactylorhiza majalis* showed negative ID values in three out of four reproductive features studied (seed length, frequency of well-developed embryos in seeds, and proportion of *in vitro* seed germination), both with and without division of inflorescence into three levels (**Figure 3**). The lowest ID values were consistently noted at the upper level of the inflorescence in *D. majalis*.

Conversely, in *D. fuchsii*, the ID estimate increased up to 0.400 in *in vitro* asymbiotic seed germination without division of inflorescence and across the three inflorescence levels (**Figure 3**). Other ID values in *D. fuchsii* were near zero (**Figure 3**).

Moderate ID was recorded for seed length, seed number per fruit, and the proportion of seeds with well-developed embryos in *D. incarnata* var. *incarnata* (**Figure 3**). PL was high, reaching 0.481 in *D. fuchsii*, 0.643 in *D. incarnata* var. *incarnata*, and the highest at 0.663 in *D. majalis*.

## 5 Discussion

### 5.1 Potential of inbreeding depression

The link between mating systems and inbreeding depression (ID) has been well-established in many studies. Smithson (2006) discovered that nectarless orchids tend to exhibit high levels of ID, which may be indicative of reduced self-fertilization (autogamy and/or geitonogamy). In orchids employing food-deceptive strategies, particularly those that are self-compatible, high ID is often observed. This is likely due to the fact that deceived pollinators typically visit only a few flowers among plants within populations, thereby promoting cross-pollination and reducing the likelihood of inbreeding (Ackerman, 1986; Dafni, 1987; Gill, 1989; Nilsson, 1992; Neiland & Wilcock, 1998; Jersáková et al., 2006; Juillet et al., 2006; Kropf & Renner, 2008; Jacquemyn & Brys, 2020). However, it appears that the impact of ID varies across different fitness traits. Smithson (2006) and Sletvold et al. (2012) noted significant ID in food-deceptive Orchidaceae, particularly in the early stages of life history, ranging from fruit set to protocorm development. Our study of *Dactylorhiza* orchids revealed that ID varied considerably among reproductive traits. In early life stages, such as seed viability, distinct patterns of ID were observed, suggesting that deceptive strategies in these orchids cannot be attributed to a single mechanism for reproductive success. In *D. fuchsii*, significant ID was evident, with outcrossed progeny demonstrating superior fitness compared to selfed progeny. This shows a strong outcrossing mating system, as described by Husband & Schemske (1996). However, the frequency of *in vitro* germinated seeds from selfed and control-pollinated flowers was similar, yet lower than that from outcross pollination. It provided only partial support for the hypothesis that inbred progeny harboring deleterious alleles are eliminated during over-fertilization or early seed development.

The observed cumulative *δ* value in *D. fuchsii* (0.366) aligns roughly with previous estimates for other species, such as *D. sambucina* (*δ* = 0.42, Nilsson, 1980), but is lower than reported by Jersáková et al. (2006), (*δ* = 0.63) and Juillet et al. (2006), (*δ* = 0.47). This value was similar to Smithson (2006) findings for nectarless orchids (*δ* = 0.32 ± 0.05) and lower than that reported by Husband & Schemske (1996) for allogamous angiosperms (*δ* = 0.53). The remarkably low ID in *D. majalis* and the absence of ID in *D. incarnata* var. *incarnata* raise intriguing questions. In *D. majalis*, strong outbreeding depression, evidenced by differences in seed length, well-developed embryos, and *in vitro* seed germination between outcross and self-progeny, was observed. However, no significant differences were noted across three inflorescence levels. *Dactylorhiza incarnata* var. *incarnata* exhibited outbreeding depression in seed germination, while hand-selfed flowers produced seeds with similar viability to naturally pollinated flowers. These results suggest that in *D. majalis* and *D. incarnata* var. *incarnata*, pollinator limitation is likely, and ID is negligible, with biparental inbreeding being more common. The negligible ID in *D. incarnata* var. *incarnata* (*δ* = 0.065) may reflect a historical prevalence of selfing, leading to a reduced genetic load and decreased inbreeding depression (Husband & Schemske, 1996). Hedrén & Nordström (2009) proposed that populations of *D. incarnata* var. *incarnata* might consist of several inbred lines. This hypothesis is supported by studies from Naczk et al. (2018) and Vallius et al. (2004). The extremely low ID in the allopolyploid *D. majalis*, potentially exacerbated by within-population relative pollination, suggests reduced fitness due to detrimental alleles at a local geographic scale (Husband et al., 2008). There is evidence suggesting a lack of selection against selfed progeny in this polyploid. It is plausible that inbred progeny harboring deleterious alleles are not eliminated during over-fertilization or early germination phases (Ortiz-Barney & Ackerman, 1999). *Dactylorhiza majalis*, an allotetrapolyploid relative to its diploid progenitors *D. incarnata* and *D. fuchsii* (Pillon et al., 2007), represents a complex taxonomic group that evolved through multiple and independent hybridization events. The genus’s taxonomic complexity is likely attributable to its migration history during glaciations and interglacial periods, as well as episodes of polyploidization. Otto & Whitton (2000) noted that taxa belonging to polyploidy, which are at or near mutation-selection equilibrium, are expected to harbor greater genetic loads than comparable diploids, potentially leading to higher levels of ID in the progenitors (Ronfort, 1999; Pannell et al., 2004).

### 5.2 Mating system and pollen limitation

A mixed mating system was observed in all studied *Dactylorhiza* taxa (Hedrén & Nordström, 2009; Ostrowiecka et al., 2019). Ostrowiecka et al. (2019) found that pollinator behavior in *D. majalis* likely promotes geitonogamy, explaining the development of selfed seeds in fruits at different levels of the inflorescence with germination potential similar to that of outcrosses within populations. Our results, particularly concerning the level of fruit set, corroborate previous studies on *Dactylorhiza* (Neiland & Wilcock, 1998; Vallius, 2000; Claessens and Kleynen, 2011). We did not confirm spontaneous autogamy in the three *Dactylorhiza* taxa, and our findings affirm that these taxa are highly dependent on pollinators, with a notable presence of pollen limitation, especially in *D. majalis*. This observation aligns with the common occurrence of pollen limitation in deceptive orchids (Tremblay, 2006; Henneresse et al., 2017). The foraging behavior of deceived pollinators likely results in the transfer of low amounts of pollen per visit, increasing the risk of pollen limitation and reducing the likelihood of receiving pollen from different individuals. Ostrowiecka et al. (2019) reported that mean visitation frequency by *Apis mellifera* was extremely low and time of visits of *A. mellifera* on inflorescences lasted from 11-40 s in studied *D. majalis.* Additionally, our data suggest that pollinator foraging behavior remained consistent across the inflorescence during the flowering season, indicating that flower position on the inflorescence did not significantly impact fitness components such as seed length, well-developed seeds, and *in vitro* asymbiotic seed germination. However, the observed decrease in seed number per fruit from the lower to the upper level of the inflorescence is typically attributed to the resource hypothesis, suggesting increased competition for resources among pollinated flowers (Devlin, 1989; Berry & Calvo, 1991; Obeso, 1993; Diggle, 1995). This decrease in seed set across the inflorescence has also been noted in *Dactylorhiza maculata* and *Epipactis helleborine*, potentially due to limited resources and morphological differences in the upper flowers (Light & MacConaill, 1998; Vallius, 2000). In summary, our study as the first revealed that fitness traits in early life stage in three *Dactylorhiza* taxa depend significantly on type of mating system, but little influence on flower position on inflorescence. These hypotheses warrant further investigation in the context of the three taxa studied.

## 6 Conclusions

Our experimental findings revealed that high ID was maintained only in the diploid, food-deceptive *D. fuchsii*, particularly at the asymbiotic *in vitro* germination stage. In contrast, ID manifested at low to moderate levels in the allotetraploid *D. majalis* and, to some extent, in its diploid ancestor *D. incarnata* var. *incarnata*, with little evidence of selection against selfed offspring. Additionally, our study showed that the mating system, rather than the position of flowers on the inflorescence, primarily influenced the quality and quantity of reproductive output. The impact of ID on the germination life stage played a significant role in the reproductive ecology of these taxa.

## Data availability statement

The original contributions presented in the study are included in the article/**Supplementary Material**. Further inquiries can be directed to the corresponding author.

## Author contributions

AW: Conceptualization, Analysis, Investigation, Methodology, Project administration, Writing – original draft, editing. BO: Investigation, Writing – original draft. EB: Investigation, Writing – original draft. EJ: Investigation, Writing – original draft. IT: Investigation, Writing – original draft. PM: Analysis with R package, Writing – original draft. All authors contributed to the article and approved the submitted version.
