# Supplementary material for: The patterns of inbreeding depression in food-deceptive Dactylorhiza orchids
Source: Front Plant Sci. 2024 Mar 25;15:1244393. doi: 10.3389/fpls.2024.1244393 (PMC10999633; doi:10.3389/fpls.2024.1244393)
Supplement: Supplementary file 1 [file Table_1.docx]

| Taxon/population/year | | | GPS location | N | | | N_IFN_ | | N_FN_ | | N_IFAS_ | | N_FAS_ | | N_IFAI_ | | N_FAI_ | | N_IFG_ | | N_FG_ | | N_IFX_ | N_FX_ | E |
| --- | --- | --- | --- | --- | --- | --- | --- | --- | --- | --- | --- | --- | --- | --- | --- | --- | --- | --- | --- | --- | --- | --- | --- | --- | --- |
| ***Dactylorhiza majalis*** | | |  |  | | |  | |  | |  | |  | |  | |  | |  | |  | |  |  |  |
| KA | 2014 | | 52°53’00’’N 23°40’29’’E | ~ 1000 | | | 5 | | 110 | | 4 | | 62 | | 5 | | 71 | | 3 | | 44 | | 5 | 105 | 0.39 |
|  | 2015 | |  | ~ 1000 | | | 5 | | 160 | | 5 | | 154 | | 5 | | 105 | | 5 | | 141 | | 5 | 142 | 0.64 |
| SKI | 2015 | | 52°49’50’’N 23°43’10’’E | ~ 200 | | | 5 | | 110 | | 5 | | 90 | | 5 | | 70 | | 5 | | 78 | | 5 | 85 | 0.56 |
|  | 2016 | |  | ~ 180 | | | 5 | | 118 | | 3 | | 47 | | 5 | | 49 | | 5 | | 67 | | 5 | 93 | 0.62 |
| SKII | 2016 | | 52°49’50’’N 23°43’10’’E | ~ 150 | | | 5 | | 124 | | 3 | | 43 | | 5 | | 90 | | 5 | | 70 | | 5 | 100 | 0.60 |
|  | 2017 | |  | ~ 120 | | | 5 | | 131 | | 5 | | 42 | | 5 | | 71 | | 5 | | 47 | | 5 | 122 | 0.59 |
| *Σ* | | | | |  | 30 | | 730 | | 25 | | 438 | | 30 | | 456 | | 28 | | 447 | | 30 | | 647 |  |
| ***D. incarnata* var. *incarnata*** | | | | |  |  | |  | |  | |  | |  | |  | |  | |  | |  | |  |  |
| MR | | 2015 | 53°47’25’’N  22°57’22’’E | 100 | | | 5 | | 172 | | 5 | | 118 | | 5 | | 103 | | 2 | | 37 | | 5 | 141 | 0.76 |
|  | | 2016 |  | 68 | | | 5 | | 130 | | 4 | | 93 | | 1 | | 20 | | 5 | | 97 | | 2 | 76 | 0.47 |
| ZB | | 2014 | 53°17’59’’N  22°35’44’’E | ~ 100 | | | 5 | | 340 | | 5 | | 327 | | 5 | | 284 | | 5 | | 203 | | 5 | 177 | 0.41 |
|  | | 2015 |  | ~ 80 | | | 5 | | 270 | | 5 | | 225 | | 5 | | - | | 5 | | 104 | | 5 | 151 | 0.58 |
| RO | | 2015 | 53°54’39’’N  22°56’32’’E | ~ 200 | | | 5 | | 166 | | 4 | | 86 | | 5 | | 96 | | 2 | | 115 | | 5 | 88 | 0.61 |
|  | | 2016 |  | ~ 156 | | | 5 | | 197 | | 2 | | 56 | | 5 | | 143 | | 5 | | 143 | | 5 | 131 | 0.78 |
|  | | | *Σ* |  | | | 30 | | 1275 | | 25 | | 905 | | 26 | | 646 | | 24 | | 519 | | 27 | 764 |  |
| ***Dactylorhiza fuchsii*** | | |  |  | | |  | |  | |  | |  | |  | |  | |  | |  | |  |  |  |
| BR | 2014 | | 52°50’59’’N  23°53’40’’E | 102 | | | 5 | | 90 | | 5 | | 106 | | 5 | | 66 | | 5 | | 122 | | 5 | 128 | 0.69 |
|  | 2015 | |  | 133 | | | 5 | | 123 | | 5 | | 90 | | 5 | | 115 | | 5 | | 106 | | 5 | 101 | 0.53 |
| CM | 2015 | | 52°41’03’’N 23°39’07’’E | 122 | | | 5 | | 205 | | 5 | | 145 | | 5 | | 90 | | 5 | | 75 | | 5 | 128 | 0.77 |
|  | 2016 | |  | 84 | | | 5 | | 110 | | 4 | | 150 | | 5 | | 127 | | 3 | | 39 | | 5 | 101 | 0.60 |
| GR | 2014 | | 53°36’28’’N  22°50’26’’E | 140 | | | 5 | | 109 | | 5 | | 110 | | 5 | | 76 | | 5 | | 83 | | 5 | 111 | 0.83 |
|  | 2015 | |  | 193 | | | 5 | | 131 | | 3 | | 55 | | 5 | | 64 | | 3 | | 43 | | 5 | 115 | 0.49 |
|  |  | | *Σ* |  | | | 30 | | 768 | | 27 | | 656 | | 30 | | 538 | | 26 | | 468 | | 30 | 684 |  |

Table S1. Characteristic of nine *Dactylorhiza majalis, D. incarnata* var. *incarnata,* and *D. fuchsii* populations in relation to locations; N – number of flowering plants; N_IFN_ – sum of flowering inflorescences in control pollination, N_IFAS_  – spontaneous autogamy, N _IFAI_  – induce autogamy, N_IFG_ – geitonogamy, and N_IFX_ – xenogamy, respectively; N_FN_ – sum of flowers in control pollination, N_FAS_  – spontaneous autogamy, N _FAI_ – induce autogamy, N_FG_ – geitonogamy, and N_IFX_ – xenogamy, respectively; E – equivalence factor (Lloyd 1980); x – an average of value; SD – standard deviation. KA – Kapitańszczyzna, SKI – Skupowo I, SKII – Skupowo II, MR – Marachy, ZB – Bagno Ławki, RO – Rospuda, BR – Browsk, CM – Cimoszewizna, GR – Grzędy population (see Fig. 1).
