# Supplementary material for: The patterns of inbreeding depression in food-deceptive Dactylorhiza orchids
Source: Front Plant Sci. 2024 Mar 25;15:1244393. doi: 10.3389/fpls.2024.1244393 (PMC10999633; doi:10.3389/fpls.2024.1244393)
Supplement: Supplementary file 2 [file Table_2.docx]

| ***Dactylorhiza majalis*** | | | | | | | | | | | | | | | | | | | |  |
| --- | --- | --- | --- | --- | --- | --- | --- | --- | --- | --- | --- | --- | --- | --- | --- | --- | --- | --- | --- | --- |
|  | control pollination/level on the inflorescence | | | | | | cross-pollination/level on the inflorescence | | | | | | selfing/level on the inflorescence | | | | T L | | |  |
|  | 1 | 2 | 3 | mean | χ^2^ (*P*) | | 1 | 2 | 3 | | mean | χ^2^ (*P*) | 1 | 2 | 3 | mean | | χ^2^ (*P*) χ^2^ (*P*) χ^2^ (*P*) | |  |
| fruit set 40.9 39.3 24.9 35.4  ±0.2 ±0.1 ±0.2 ±0.1 | | | | | 1.0 ns | 89.2  ±15.0 | | 91.0  ±17.5 | 89.9  ±21.0 | | 90.0  ±17.8 | 0.01 ns | 91.6  ±0.1 | 93.8  ±0.1 | 90.6  ±0.1 | 92.0  ±12.9 | | 0.01 ns 53.97 *** 0.43 ns | |  |
| number of seeds per fruit | 4759.0 ±403.7 | 3679.9 ±347.3 | 3525.1 ±358.6 | 3999.8 ±225.0 | 3.13 * | | 3671.0 ±528 | 3231.3 ±543 | 1889.8 ±257 | | 2908.6 ±278.1 | 6.79 * | 2992.2 ±300.0 | 2664.9 ±213.0 | 2334.7 ±282.0 | 2675.9 ±156.0 | | 1.48 ns 27.6 *** 7.91 ** | |  |
| seed length (mm) | 0.688 ±0.02 | 0.662 ±0.01 | 0.677 ±0.01 | 0.678 ±0.01 | 1.35 ns | | 0.579 ±0.01 | 0.566 ±0.01 | 0.588 ±0.02 | | 0.578 ±0.01 | 1.65 ns | 0.662 ±0.01 | 0.651 ±0.01 | 0.802 ±0.01 | 0.703 ±0.01 | | 1.40 ns 41.00 *** 6.44 ns | |  |
| seeds with well-developed embryos (%) | 53.6 ±7.0 | 51.9 ±6.9 | 50.8 ±6.8 | 51.7  ±3.9 | 0.11 ns | | 30.4 ±6.1 | 32.6 ±8.3 | 15.6 ±5.6 | | 26.2 ±4.1 | 0.97 ns | 44.2 ±5.3 | 32.9 ±4.6 | 42.6 ±5.0 | 39.8 ±2.9 | | 2.68 ns 12.8* 1.42 ns | |  |
| in vitro asymbiotic seed germination (%) | 28.3 ±4.4 | 26.2 ±4.2 | 27.3 ±4.3 | 26.4 ±0.4 | 1.18 ns | | 30.7 ±4.5 | 23.9 ±5.0 | 21.33 ±4.8 | | 25.2 ±2.8 | 4.22 * | 30.0 ±3.7 | 30.2 ±3.8 | 28.1 ±3.5 | 29.3 ±2.1 | | 0.26 ns 3.91 ns 3.30 ns | |  |
| ***Dactylorhiza incarnata* var. *incarnata*** | | | | | | | | | | | | | | | | | | | |  |
|  | control pollination/level on the inflorescence | | | | | | cross-pollination/level on the inflorescence | | | | | | selfing/level on the inflorescence | | | | | | |  |
|  | 1 | 2 | 3 | mean | χ^2^ (*P*) | | 1 | 2 | 3 | | mean | χ^2^ (*P*) | 1 | 2 | 3 | mean | | χ^2^ (*P*) χ^2^ (*P*) χ^2^ (*P*) | |  |
| fruit set | 34.0  ±0.3 | 33.4  ±0.3 | 24.2  ±0.3 | 30.5  ±0.3 | 4.0 ns | | 74.9  ±0.1 | 81.4  ±0.1 | 82.1  ±0.1 | | 80.2  ±21.0 | 0.57 ns | 98.6  ±0.1 | 95.6  ±0.1 | 92.6  ±0.1 | 95.6  ±11.0 | | 0.00 ns 66.40 *** 0.27 ns | |  |
| number of seeds per fruit | 10834.1 ±801.5 | 8023.2 ±714.4 | 5411.2 ±467.7 | 8089.0 ±373.2 | 21.91 *** | | 11707.8±1087.0 | 10570.5±662.7 | 6329.2 ±801.6 | | 9535.8 ±634.9 | 10.04 ** | 9599.3 ±662.7 | 8285.5 ±640.4 | 6172.9 ±505.4 | 7993.8 ±472.4 | | 18.03 ** 5.82 ns 53.23 *** | |  |
| seed length (mm) | 0.591 ±0.01 | 0.590 ±0.01 | 0.582 ±0.01 | 0.588 ±0.01 | 0.90 ns | | 0.589 ±0.10 | 0.557 ±0.01 | 0.545 ±0.12 | | 0.564 ±0.01 | 1.11 ns | 0.545 ±0.01 | 0.568 ±0.01 | 0.514 ±0.01 | 0.545 ±0.00 | | 4.32 * 10.80 *** 1.36 ns | |  |
| seeds with well-developed embryos (%) | 55.1 ±4.7 | 55.1 ±5.8 | 47.8 ±6.8 | 52.8 ±3.3 | 0.40 ns | | 48.1 ±4.8 | 52.3 ±3.5 | 48.2 ±9.8 | | 49.6 ±3.6 | 0.48 ns | 40.2 ±4.8 | 33.3 ±5.2 | 46.1 ±5.7 | 40.2 ±3.0 | | 0.67 ns 7.31 * 0.05 ns | |  |
| in vitro asymbiotic seed germination (%) | 15.5 ±4.8 | 14.1 ±2.8 | 11.8 ±4.1 | 13.8 ±1.0 | 0.39 ns | | 11.8 ±3.0 | 14.7 ±2.9 | 10.9 ±2.1 | | 10.2 ±2.1 | 0.11ns | 11.4 ±3.0 | 14.2 ±2.9 | 10.1 ±2.1 | 11.9 ±1.5 | | 0.58 ns 2.91 ns 0.20 ns | |  |
| ***Dactylorhiza fuchsii*** | | | | | | | | | | | | | | | | | | | |  |
|  | control pollination/level on the inflorescence | | | | | | cross-pollination/level on the inflorescence | | | | | | selfing/level on the inflorescence | | | | | | |  |
|  | 1 | 2 | 3 | mean | χ^2^ (*P*) | | 1 | 2 | 3 |  | mean | χ^2^ (*P*) | 1 | 2 | 3 | mean | | χ^2^ (*P*) χ^2^ (*P*) χ^2^ (*P*) | |  |
| fruit set | 46.9  ±0.6 | 42.7  ±0.6 | 31.3  ±0.6 | 40.0  ±0.6 | 0.57 ns | | 84.8  ±0.1 | 87.9  ±0.1 | 83.2  ±0.1 | | 85.0  ±20.8 | 0.01 ns | 91.8  ±0.1 | 92.3  ±0.1 | 82.6  ±0.1 | 88.9  ±18.9 | | 0.01 ns 61.90 *** 2.12 ns |  | |
| number of seeds per fruit | 3109.9 ±318.9 | 2318.0 ±245.7 | 1616.1 ±182.2 | 2332.4 ±166.8 | 11.32 ** | | 1788.6 ±293.6 | 1708.6 ±163.0 | 1386.8 ±108.5 | | 1629.4  ±117.1 | 1.32 ns | 2120.2 ±257.9 | 1836.2 ±182.3 | 1474.0 ±169.4 | 1810.9 ±120.7 | | 3.16 ns 18.75 ** 10.88** |  | |
| seed length (mm) | 0.798 ±0.01 | 0.764 ±0.01 | 0.783 ±0.01 | 0.782 ±0.02 | 1.53 ns | | 0.727 ±0.02 | 0.730 ±0.01 | 0.723 ±0.01 | | 0.729 ±0.02 | 0.73 ns | 0.725 ±0.01 | 0.753 ±0.01 | 0.707 ±0.01 | 0.728 ±0.01 | | 10.70 ** 16.51 *** 2.88 ns | |  |
| seeds with seeds with well-developed embryos (%) | 59.9 ±4.7 | 60.6 ±5.0 | 59.0 ±5.0 | 59.8 ±2.7 | 0.61 ns | | 39.2 ±3.7 | 51.0 ±2.9 | 46.8 ±6.0 | | 45.4 ±2.5 | 1.04 ns | 43.3 ±4.7 | 47.5 ±4.5 | 43.5 ±5.3 | 44.8 ±2.7 | | 1.87 ns 6.09 ** 4.38 ns | |  |
| in vitro asymbiotic seed germination (%) | 64.9 ±5.5 | 38.5 ±4.2 | 15.9 ±4.1 | 39.4 ±3.5 | 20.89 *** | | 58.5 ±6.1 | 63.5 ±4.5 | 62.0 ±2.2 | | 61.3 ±2.6 | 0.60 ns | 40.2  ±3.5 | 39.4 ±3.2 | 33.5 ±3.5 | 37.6 ±2.0 | | 3.50 ns 21.17 *** 9.66** | |  |

Table S2. Characteristic of fruit set, quantity and quality of seeds among control- (open), cross-, and self-pollination treatments (induced autogamy and geitonogamy, together) and among three levels of inflorescence (lower, 1; middle, 2; and upper 3) among treatments of *Dactylorhiza majalis*. *D. incarnata* var. *incarnata*, and *D. fuchsii*; ± SE (standard error); ***, *p* < 0.001. **, *p* < 0.01. *, *p* < 0.05, ns – not significant. T – differences between hand pollination treatments (Kruskal-Wallis test), L – differences between levels in pollination treatments (Kruskal-Wallis test).
